# Action-driven contrastive representation for reinforcement learning

**DOI:** 10.1371/journal.pone.0265456

**Published:** 2022-03-18

**Authors:** Minbeom Kim, Kyeongha Rho, Yong-duk Kim, Kyomin Jung

**Affiliations:** 1 Graduate School of Artificial Intelligence, Seoul National University, Seoul, Republic of Korea; 2 Department of Electrical and Computer Engineering, Seoul National University, Seoul, Republic of Korea; 3 Defense AI Technology Center, Agency for Defense Development, Daejeon, Republic of Korea; Hanyang University, KOREA, REPUBLIC OF

## Abstract

In reinforcement learning, reward-driven feature learning directly from high-dimensional images faces two challenges: *sample-efficiency* for solving control tasks and *generalization* to unseen observations. In prior works, these issues have been addressed through learning representation from pixel inputs. However, their representation faced the limitations of being vulnerable to the high diversity inherent in environments or not taking the characteristics for solving control tasks. To attenuate these phenomena, we propose the novel contrastive representation method, *Action-Driven Auxiliary Task (ADAT)*, which forces a representation to concentrate on essential features for deciding actions and ignore control-irrelevant details. In the augmented state-action dictionary of ADAT, the agent learns representation to maximize agreement between observations sharing the same actions. The proposed method significantly outperforms model-free and model-based algorithms in the Atari and OpenAI ProcGen, widely used benchmarks for sample-efficiency and generalization.

## Introduction

Reinforcement learning (RL) has achieved state-of-the-art performance on a variety of sequential decision tasks [1]. With a lot of trials-and-errors, agents obtain competent policies achieving human-level control in complex tasks. Despite the successes in simulation games, however, RL has faced the limitation that numerous trials-and-errors are essential for learning. In real world, collecting such an enormous amount of trials is time-consuming and requires large amounts of resources. Furthermore, the unexpected factors in new environments can yield test-performance decay. Therefore, sample-efficiency and generalization capability in RL are emerged as challenging tasks.

In general, state representation from raw pixel inputs contributes to efficient exploration and robustness to zero-shot observations. This intuition has been proven experimentally in various environments through comparisons between state-based exploration and learning in high dimensional observations [2, 3]. Therefore, learning representation is considered as crucial apparatus for sample-efficiency and generalization. To address learning representation in RL, various approaches have been proposed in the literature. Broadly, there are three mainstreams on Auxiliary tasks: (1) reconstructing pixel-inputs, (2) World Model building predictive models of environments, and (3) contrastive representation learning.

**Fig 1:**
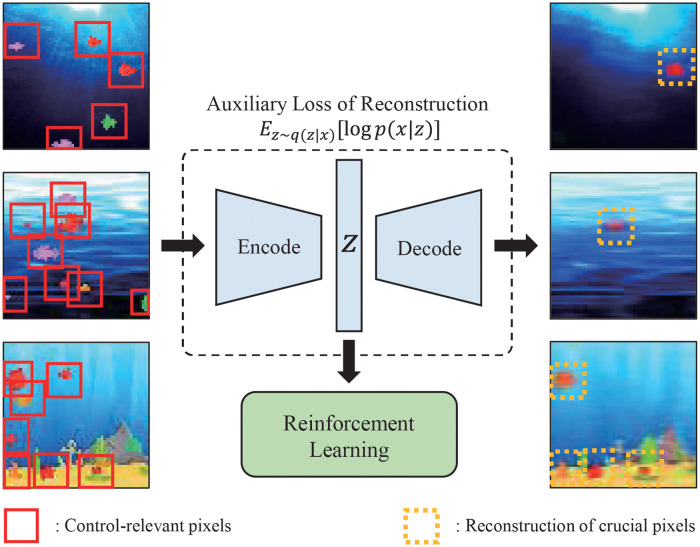
Reconstruction of auto-encoder based representation. This is the Bigfish of ProcGen environment, which has various wallpaper patterns in the background. Under the auxiliary task of reconstructing original inputs, left images are pixel-inputs and right are reconstructions of their representations. It shows a problem that if there are various control-irrelevant details in wallpaper pixels, the encoder first learns abstraction about irrelevant details for gameplay instead of the essential pixels (i.e., fish in this example). Our proposal was motivated by this issue.

Methodologies with reconstruction errors [4, 5] and World Model [6] yielded breakthroughs in representation mechanism, gaining various advantages such as sample-efficiency, the efficacy of exploration, and domain transfer [7–9]. Yet, they might suffer from difficulties when facing complex environments or environments with a lot of control-irrelevant visual information, as shown in Fig 1. To overcome the limitation of these methods, Contrastive Unsupervised Representations for Reinforcement Learning (CURL) proposed an auxiliary task maximizing accordance between different augmented versions of the same images [10]. CURL gains significant sample-efficiency, outperforming existing model-free and model-based methodologies. However, only with the auxiliary task of CURL, the agent considers only image augmentation-invariant features. It does not suffice to distinguish whether visual information is control-relevant or not. Under those circumstances, [11, 12] extend auxiliary tasks of CURL to consider accordance between temporally consecutive observations or similar returns. [13] points out the same problem as our research and represents control-relevant objects through a learned world model. However, looking at previous researches, their auxiliary tasks too focus on environment-specific accordance. For various downstream tasks, agents should extract intrinsic representation relevant to overall control problems. To learn intrinsic representation, the auxiliary task needs to rethink ‘actions decided by the observations’. The rich history of ‘state-action pairs’ contains the intuitions obtained by the agent from numerous interactions with the environment. Therefore, auxiliary task should be reformed to leverage state-action pairs as self-made labels.

This paper proposes a novel auxiliary task *Action-Driven Auxiliary Task* (ADAT). The proposed method adopts pseudo-supervised contrastive learning through instance discrimination about the states sharing the same actions. Our hypothesis is straightforward: representation can capture the motivational key-features for deciding an action and ignore the control-irrelevant information through contrastively aggregated states labeled by actions. This intuition is understood visually with our experiment of saliency map [14]. Also, we suggest Unbiased Sampling module for attaching ADAT to existing RL algorithms. Consequently, *Action-Driven Contrastive Representation for Reinforcement Learning* is designed based on the proposed methodologies. The effect of improving performance in sample-efficiency and robustness compared to existing baselines is verified by conducting experiments. In Atari Games, widely used benchmarks for measuring sample-efficiency, our method achieves state-of-the-art in 15 out of 26 games and outperforms human performance on five games. Moreover, the agents in ProcGen Games show dramatically improved generalization ability to unseen observations. Finally, the saliency map experiment is conducted to visually understand what ADAT agents concentrate on.

The contributions of this paper are summarized as: (1) ADAT, the novel auxiliary task, dedicated to solving control tasks; (2) Unbiased Sampling for ADAT to be compatible with off-policy algorithms; (3) empirical demonstration of superior sample-efficiency and generalization in Atari Games and OpenAI ProcGen, and visual understanding of action-supervision’s efficacy with saliency map.

## Background

Simply supervision-driven or reward-driven features have struggled with real-world problems for downstream tasks [15]. For enriching features without external supervision, contrastive learning defines an instance discrimination task [16], where the positive key should be distinguished from the negatives according to the given query. CURL [10] learns representation in this manner. An input image goes through ‘the augmentation of random crop’ and results in a query *q* and the positive key *k*_+_. Denote *K* = {*k*_+_}∪{*k*_1_, *k*_2_, …, *k*_*N*−1_}, the negative keys sampled from the CURL’s replay buffer. The dictionary is looked-up as $D=\{f_{k}(T(k))|k\in K\}$, where T is the augmentation and *f*_*k*_ is the key encoder. Through the query encoder *f*_*q*_, the representation of the query and keys are measured as a pairwise similarity. The encoded query is used for policy optimization, such as DQN Rainbow [17] and Soft Actor-Critic [18]. In the process of maximizing an agreement between the query and the positive key, *f*_*q*_ is updated by contrastive loss and policy loss of interactions with environments. On the other hand, *f*_*k*_ is only trained by momentum contrast update [19] as below:

$$\begin{array}{c} \begin{array}{c} \theta_{f_{k}}=m\times \theta_{f_{k}}+\left(1-m\right)\times \theta_{f_{q}}. \end{array} \end{array}$$ (1)

To respect the relative similarity of embeddings, CURL measures pairwise similarity as contrastive predictive coding [20]. This method was proposed for an encoder to measure the underlying shared structure contrastively rather than trivial information by inserting a bilinear product into measuring the pairwise similarity of a query and keys:

$$\begin{array}{c} \begin{array}{c} f\left(q,k\right)=\text{exp}\left(q^{T}Wk\right), \end{array} \end{array}$$ (2)

Representation features predict far in the contrastive measuring, extracting meaningful agreement. *W* can be the linear transformation or non-linear neural networks for the bilinear product of query *q* and key *k*. For calculating gradients of dictionary {*k*_1_, *k*_2_, *k*_3_, …, *k*_*n*_} for each query *q*, InfoNCE loss was proposed for segregating one positive keys *k*_+_ and *N* − 1 negative keys as:

$$\begin{array}{c} \begin{array}{c} L_{q}=\text{log}\frac{\text{exp}(q^{T}Wk_{+})}{\text{exp}\left(q^{T}Wk_{+}\right)+\sum_{i=1}^{N-1}\text{exp}\left(q^{T}Wk_{i}\right)}. \end{array} \end{array}$$ (3)

Therefore, Contrastive Unsupervised Representations for Reinforcement Learning adopted InfoNCE loss, discriminating one positive key for each query, a different image-augmented observation of query image. Through an end-to-end contrastive representation learning mechanism, CURL has shown state-of-the-art performance in representation learning across continuous and discrete control benchmark tasks. Recently, InfoNCE loss has been reformed to reinterpret self-supervised learning for a variety of purposes [21].

## Method

### Action-driven auxiliary task

**Fig 2:**
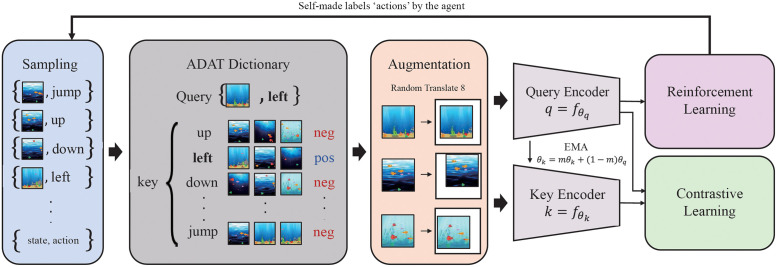
The overall framework of ADAT. State-action pairs are sampled from a history of interactions. The dictionary is built with states by action types, labeling keys based on the query’s action. Through augmentation ‘Random-Translate’ [22], query is encoded for reinforcement learning and query-key pairs are encoded for contrastive learning [20]. Only the query encoder learns from contrastive loss, and the key encoder is trained as Momentum Update [19].

We propose the novel auxiliary task for learning intrinsic representation about solving control tasks. To represent the intrinsic features that determine the action, an auxiliary task needs to utilize the knowledge of the agent who has accumulated intuition about the environment through a number of interactions. The rich history of interactions will be of great help in identifying only crucial relevance between visual information and actions. Our auxiliary task, Action-Driven Auxiliary Task (ADAT), forces learned representation to become invariant about control-irrelevant pixels and sensitive to essential pixels for control tasks. From the history of interactions such as the replay buffer or a minibatch rolled out by runners, ADAT samples state-action pairs {*x*, *a*} to build a dictionary consisting of the query {*x*_*q*_, *a*_*q*_} and keys $\left\{\left\{x_{k_{1}},a_{k_{1}}\right\},\left\{x_{k_{2}},a_{k_{2}}\right\},\left\{x_{k_{3}},a_{k_{3}}\right\},\ldots ,\left\{x_{k_{n}},a_{k_{n}}\right\}\right\}$ as shown in Fig 2. Randomly augmented states $T(x_{k})$ (e.g., T: translate [22]) are encoded into representation $f_{k}\left(T\left(x_{k}\right)\right)$ and become positive keys of the dictionary if sharing the same actions with the query. The projected pairwise similarity is measured as a bilinear product with contrastive predictive coding [20] which can help capture meaningful structures other than irrelevant minors. In each minibatch, this dictionary is looked up as pseudo-supervised contrastive learning to maximize agreement between the query and its positive keys. Unlike CURL, there are multiple positive keys per query because there is not only one state that the agent made the same choice in history. Therefore, multi-positive keys should be matched with each query in the dictionary. We employ the log-summation loss with binary cross-entropy of sigmoid classifier other than InfoNCE, interpreted as cross-entropy of softmax classifier.

$$\begin{array}{c} \begin{array}{c} L^{ADAT}=\sum_{i\in I}\sum_{j\in J}L_{i,j}=\sum_{i\in I}\sum_{j\in J}\ell \left(z_{i}^{T}z_{j},label_{i,j}\right),\\ \ell \left(z_{i}^{T}z_{j},label_{i,j}\right)=\{\begin{array}{cc} -log\frac{\text{exp}(z_{i}^{T}z_{j})}{\text{exp}(z_{i}^{T}z_{j})+1} & label_{i,j}=1,\\ -log\frac{1}{\text{exp}(z_{i}^{T}z_{j})+1} & label_{i,j}=0, \end{array} \end{array} \end{array}$$ (4)

where $z_{i}=Proj\left(f\left(T\left(x_{i}\right)\right)\right)$ is the linear projection of the augmented pixel-input’s representations. *I* and *J* are the sets of indices for all the elements augmented differently in the dictionary. *ℓ* is log-loss, which measures agreement between two representations. *label*_*i*,*j*_ is a pseudo-label that means if the agent chose the same action in both states, it becomes a positive label. Product of linear projections $z_{i}^{T}z_{j}$ can be reformed as a bilinear product of query and key *q*^*T*^*Wk*, contrastive predictive coding for capturing meaningful shared structures. The sigmoid function activates pairwise similarity $z_{i}^{T}z_{j}$ and $L^{ADAT}$ aggregates *ℓ*, binary cross-entropy loss of all sigmoid pairwise similarities in a dictionary.

**Algorithm 1** ADAT’s main learning algorithm

**Input:** batch size N, momentum m,

*θ*_*query*_ of query encoder *f*_*q*_, *θ*_*key*_ of key encoder *f*_*k*_,

linear projection *g*, set of random augmentations T

**for** sampled batch ${\left\{x_{k},a_{k}\right\}}_{k=1}^{N}$

 **for all** *k* ∈ {1, …, *N*} **do**

  draw random augmentation t∼T, $t^{\prime }∼T$

  # query inference

  ${\tilde{x}}_{2k-1}=t\left(x_{k}\right)$

  $h_{2k-1}=f_{q}\left({\tilde{x}}_{2k-1}\right)$    ⊳ representation of query

  $z_{2k-1}=g\left({\tilde{h}}_{2k-1}\right)$      ⊳ projection

  *a*_2*k*−1_ = *a*_*k*_

  # key inference

  ${\tilde{x}}_{2k-1}=t^{\prime }\left(x_{k}\right)$

  $h_{2k}=f_{k}\left({\tilde{x}}_{2k-1}\right)$    ⊳ representation of key

  $z_{2k}=g\left({\tilde{h}}_{2k}\right)$      ⊳ projection

  *a*_2*k*_ = *a*_*k*_

 **end for**

 **for all** *i* ∈ {1, 3, 5, …, 2*N* − 1} and *j* ∈ {2, 4, 6, …, 2*N*} **do**

  $s_{i,j}=1/\left(1+e^{-z_{i}^{T}z_{j}}\right)$    ⊳ sigmoid pairwise similarity

  $m_{i,j}=\{\begin{array}{cc} 0 & a_{i}\neq a_{j},\\ 1 & a_{i}=a_{j}, \end{array}$      ⊳ pseudo labeling

  $L_{i,j}=-m_{i,j}\times \text{log}\;s_{i,j}-\left(1-m_{i,j}\right)\times \text{log}(1-s_{i,j}$)  ⊳ binary cross entropy loss

 **end for**

 update *f*_*q*_ and *g* to minimize $\sum L_{i,j}$

 *θ*_*key*_ = *m* × *θ*_*key*_ + (1 − *m*) × *θ*_*query*_      ⊳ MoCo update

**end for**

**return** *θ*_*query*_    ⊳ for policy training phase

With this novel auxiliary task, the contrastive learner gives more attention to crucial features to decide the action. ADAT is the attachable learning representation module which can be plugged into both off-policy and on-policy algorithm. In our experiments, we attach ADAT to Rainbow DQN [17] and Proximal Policy Optimization(PPO) [23] for proving each performance improvement on off-policy and on-policy version.

### Implementation on existing baselines

**Fig 3:**
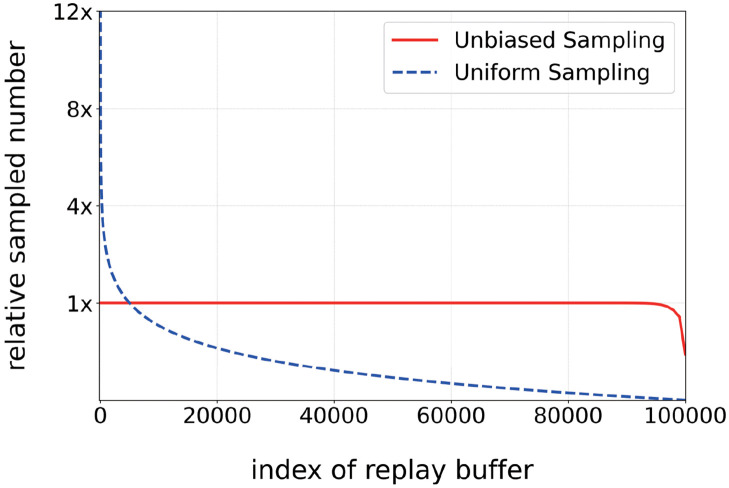
The sampling-efficacy following by two methods. By extracting twice the amount and sorting them, all elements are used equally for learning. Unbiased Sampling prevents unwise early records from being leveraged much more than rich labels.

ADAT can be compatible with both on and off-policy algorithms. The Off-policy baseline with ADAT adopts DQN Rainbow [17] as a framework for policy optimization and Momentum Contrast Update [19] as a mechanism of learning representation with the replay buffer. ADAT Rainbow builds upon the successful approach by CURL Rainbow. There are two major differences between ADAT Rainbow and CURL Rainbow. The first is a change in ‘action-driven’ contrastive representation, and the other one is *Unbiased Sampling*. For a dictionary of ADAT, actions are self-motivated supervision that the learning agents answered. Therefore, low-quality foolish answers are in the front part of the queue in the replay buffer. When sampling pseudo-labels from a uniform distribution in the ADAT dictionary, the earlier sample determined by the naive actor would be used more than the one labeled by the smarter actor as shown in Fig 3, even with prioritized experience replay, hard-converged foolish answers from less trained agents would be before consistent pairs.

Therefore, we propose Unbiased Sampling. This is a straightforward module that does not cost computationally in the total algorithm. It only takes twice as many samples as the planned minibatch size from a uniform distribution and just selects the most recent samples by the batch size from the queue. As shown in Fig 3, most elements in the replay buffer have been uniformly leveraged from unbiased sampling during the whole training time. Unbiased Sampling follows very cheap time complexity (worst case quadratic of ADAT’s batch size), so there is a little time delay in the whole training time. On our Atari Setting with ADAT Rainbow, low-quality pseudo-labels are used up to 12 times more than without unbiased sampling. It is expected that ADAT with unbiased sampling gains performance improvement by leveraging high-quality self-made labels more and raising the efficacy of sampling.

For the on-policy version with ADAT, we adopted PPO as a policy learning algorithm. Unlike off-policy algorithms using a replay buffer to get the training data, on-policy RL algorithms like PPO get the samples rolled out by the current policies. Therefore, ADAT is free from sample imbalance problems. Since the training batch size of PPO is much larger than that of ADAT in our experiments, we randomly sampled data from the training minibatches of PPO for contrastive learning. We compared our method with only reward-driven PPO to validate the robustness on unseen observations.

## Results and discussion

### Evaluation and implementation details

**Fig 4:**
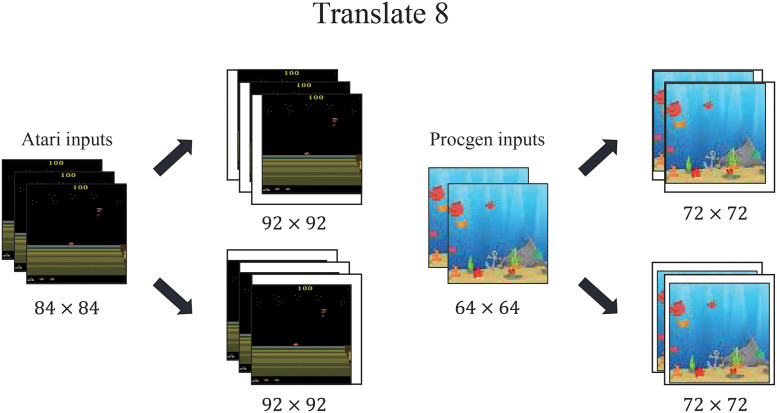
The frame-stacked inputs. For Atari agents, four sequentially frame-stacked observations are translated from 84x84 to 92x92 pixels with zero pads. Likewise, for ProcGen agents, two frame-stacked observations are translated from 64x64 to 72x72 pixels [22].

If the agent can discern the essentials for control between a lot of visual information, the significant improvement can be guaranteed on sample-efficiency for optimizing policy and generalization about robustness for zero-shot observation with similar structures. The purpose of this experiment is to measure them and to understand the proposed intuition visually. As a benchmark for sample-efficient gameplay, ADAT was employed broadly in various simulations in Atari Games. For measuring generalization capability, ProcGen Games [24] were adopted for estimating the contribution of ADAT. Then, we captured a saliency map of policies to understand which pixel information the agent focused on throughout the whole input image. By measuring how much the policies fluctuate depending on the perturbation for each pixel, we validated that our auxiliary task helped agents to concentrate more on essential pixels for decisions. Tables 1 and 2 and Fig 4 gives detailed hyperparameters for reproducing results.

**Table 1:** Hyperparameters for Atari Games 100K.

Hyperparameter	Value
Translate	True
Data Augmentation	Translate 8
Replay buffer size	100000
Training frames	400000
Training steps	100000
Stacked frames	4
Action repeat	4
Replay period every	1
Q network: channels	32, 64
Q network: filter size	5 × 5, 5 × 5
Q network: stride	5, 5
Q network: hidden units	256
EMA Momentum *τ*	0.001
Non-linearity	ReLU
Reward Clipping	[−1, 1]
Multi step return	20
Minimum replay size for sampling	1600
Max frames per episode	108K
Update	Distributional Double Q
Target Network Update Period	every 2000 updates
Support-of-Q-distribution	51 bins
Discount *γ*	0.99
Batch Size	32
Optimizer	Adam
Optimizer: learning rate	0.0001
Optimizer: *β*1	0.9
Optimizer: *β*2	0.999
Optimizer *ϵ*	0.000015
Max gradient norm	10
Exploration	Noisy Nets
Noisy nets parameter	0.1
Priority exponent	0.5
Priority correction	0.4 → 1

Across every 26 games, this setting was used the same. For Atari100K, we wanted to show that the performance is improved only by the action-driven auxiliary task. Therefore, the same experiment was conducted by importing the CURL Rainbow official code [10] as it is and modifying the contrastive learning phase.

**Table 2:** Hyperparameters for OpenAI Procgen.

Hyperparameter	Value
Translate	True
Image size	(64, 64)
Data Augmentation	Translate 8
Training frames	80000000
Training steps	40000000
Stacked frames	2
Action repeat	2
Encoder: residual blocks	3
Encoder: channels	16, 32, 32
Encoder: filter size	3 X 3, 3 X 3, 3 X 3
Encoder: strides	2, 2, 2
Encoder: latent dimension	50
Hidden units for policy and value	256
Non-linearity	ReLU
Update	PPO
Reward Clipping	[−1, 1]
Discount *γ*	0.99
Generalized Advantage Estimation λ	0.95
PPO clip range	0.2
PPO minibatches	8
PPO minibatch size	2048
Batch size for ADAT	32
ADAT updates per PPO update	1
Value loss coefficient	0.01
Optimizer	Adam
Optimizer: learning rate	0.0005
Optimizer: *β*1	0.9
Optimizer: *β*2	0.999
Optimizer *ϵ*	0.000015
Max gradient norm	0.5

For ProcGen experiments, we set the hyperparameters almost same with suggested in [24], except using data augmentation(translate) and framestack. Additionally, we adopt the residual convolutional neural network architecture used in IMPALA as the encoder with some modifications. For instance, we set the latent space dimension as 50 and add a single MLP layer with 256 hidden units to calculate policy and value logits. Then, the encoder is trained using the PPO and ADAT with this hyperparameter setting.

### Baselines

The existing baselines that are adopted for comparison with our methods are as follows,

- **Rainbow DQN** [17] is an enhanced DQN that aggregates various techniques for stabilizing RL networks into a single learner.
- **SimPLe** [9] trains the world model by self-supervised representation learning with observations collected from real environments. Then, the world model learns policy in the RL phase and gains sample-efficiency.
- **OTRainbow** [25] trains rainbow DQNs taking extra updates for sample-efficiency with repetitive samples in replay buffer, which is an advantage of DQNs
- **EFF.Rainbow** [26] suggests novel hyperparameters tuning methods for rainbow DQN’s data-efficient learning.
- **PPO** [23] is a widely used benchmark for years, suggesting novel clipped surrogate objective loss for monotonous improvements by bounded policy updates.
- **CURL** [10] leverages contrastive representation learning for sample-efficiency. Its auxiliary task learns to match different augmented versions of the same images. This self-supervision gains improved sample-efficiency, and this paper enhances it as ‘self-supervision with actions history’, outperforming existing representation methodology.

### Sample-efficiency

Atari Games were benchmarked at 100k interactions (Atari100k), which frequently have appeared as the benchmark for sample-efficiency. Rainbow DQN [17], SimPLe [9], OTRainbow [25], Efficient Rainbow [26], CURL and human scores have been baselines to show how sample-efficient our algorithm is compared to widely used representation methods. Performance of each algorithm is evaluated after 100k timesteps (400K frames, frameskip of 4) of interactions between agents and the 26 Atari Games, equivalent to two hours of gameplay. As shown in Table 3, we can empirically define the pure contribution of our novel auxiliary task ‘ADAT’ as score improvement of ADAT Rainbow over CURL Rainbow. ADAT Rainbow has shown 1.1x mean human-normalized score (HNS) gains in 100k interactions over CURL. Furthermore, ADAT+, which means ADAT with unbiased sampling gained a 1.24x higher HNS score than CURL. Not only compared to CURL, but it has also been state-of-the-art in 15 out of 26 Atari Games, surpassing human performance on five games Jamesbond (2.18HNS), Krull (1.78HNS), Road_Runner (1.60HNS), Assualt (1.01HNS) and Freeway (1.00HNS). ADAT+ Rainbow achieved the mean HNS of 47.2%, while 22.2%, 28.5%, 40.4%, 28.5% and 38.1% for Rainbow, OTRainbow, SimPLe, Efficient Rainbow and CURL. Therefore, the experimental result proves that action-driven supervision contributes to the improvement of existing contrastive representation learning methodologies. In addition, the dramatic performance improvement seen in ADAT+ confirms that the unbiased sampling module reliably solves the low-quality action labels issue.

**Table 3:** Comparison of sample efficiency.

GAME	Human	Rainbow	SimPLe	OTRainbow	EFF.Rainbow	CURL	ADAT	ADAT+
ALIEN	7127.7	318.7	616.9	824.7	739.9	558.2	953.8	**1029.7**
AMIDAR	1719.5	32.5	88.0	82.8	**188.6**	142.1	146.1	147.3
ASSAULT	742.0	231	527.2	351.9	431.2	600.6	689.5	**749.4**
ASTERIX	8503.3	243.6	1128.3	628.5	470.8	734.5	808	**864**
BANK HEIST	753.1	15.55	34.2	**182.1**	51.0	131.6	128.5	164
BATTLE ZONE	37187.5	2360.0	5184.4	4060.6	10124.6	14870.0	17160	**21240**
BOXING	12.1	-24.8	**9.1**	2.5	0.2	1.2	0.6	0.4
BREAKOUT	30.5	1.2	**16.4**	9.84	1.9	4.9	5.2	4.5
CHOPPER COMMAND	7387.8	120.0	**1246.9**	1033.33	861.8	1058.5	1151	1106
CRAZY CLIMBER	35829.4	2254.5	**62583.6**	21327.8	16185.3	12146.5	18022	21240
DEMON ATTACK	1971.0	163.6	208.1	711.8	508.0	817.6	609.8	**851.9**
FREEWAY	29.6	0.0	20.3	25.0	27.9	26.7	29.3	**29.7**
FROSTBITE	4334.7	60.2	254.7	231.6	866.8	1181.3	1838.4	**1943.2**
GOPHER	2412.5	431.2	771.0	**778.0**	349.5	669.3	634	601.2
HERO	30826.4	487	2656.6	6458.8	6857.0	6279.3	6114.2	**7259.2**
JAMESBOND	302.8	47.4	125.3	112.3	301.6	471.0	491	**635.7**
KANGAROO	3035.0	0.0	323.1	605.4	779.3	872.5	**1120**	956.9
KRULL	2665.5	1468	**4539.9**	3277.9	2851.5	4229.6	3675.9	3502.9
KUNG FU MASTER	22736.3	0.0	17257.2	5722.2	14346.1	14307.8	13767	**19146**
MS PACMAN	6951.6	67	**1480.0**	941.9	1204.1	1465.5	1144.8	1075
PONG	14.6	-20.6	**12.8**	1.3	-19.3	-16.5	-15.9	-15.1
PRIVATE EYE	69571.3	0	58.3	100.0	97.8	218.4	250	**388**
QBERT	13455.0	123.46	1288.8	509.3	1152.9	1042.4	1303.6	**1578**
ROAD RUNNER	7845.0	1588.46	5640.6	2696.7	9600.0	5661.0	9711	**12508**
SEAQUEST	42054.7	131.69	**683.3**	286.92	354.1	384.5	370.2	251.6
UP N DOWN	11693.2	504.6	3350.3	2847.6	2877.4	2955.2	3286	**3597.8**
Mean HNS	100.0%	22.2%	40.4%	26.4%	28.5%	38.1%	41.0%	**47.2%**

Atari scores of ADAT Rainbow and other baselines of 26 Atari Games benchmark achieved after 100K interactions. The average score over five random seeds. Improvements over baselines are measured as relative Human Normalized Score(HNS).

### Generalization

**Fig 5:**
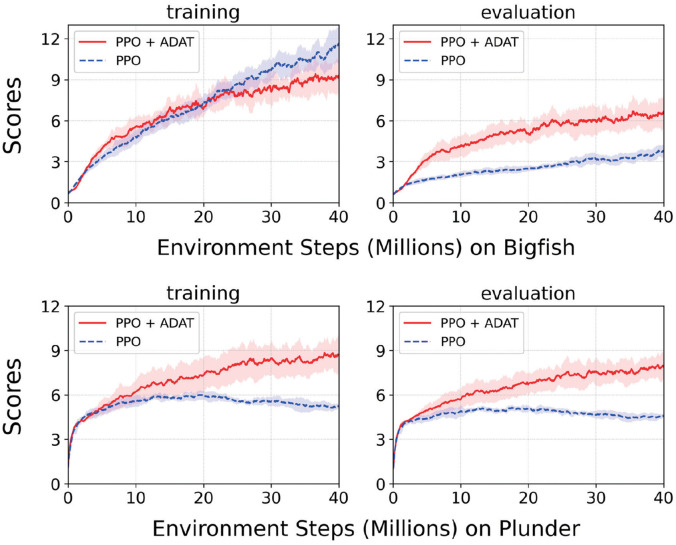
Comparison of generalization capability. Bigfish and Plunder Games in OpenAI Progen. 200-levels trained agents were evaluated on 100,000 levels. On-policy ADAT leveraged the samples rolled out by PPO policy. A difference in both frameworks is contrastive representation learning through ADAT. This figure reports the average score and standard deviation over five random seeds.

Generalization is the capability of coping with unseen observation with a similar structure. OpenAI ProcGen is a benchmark with tremendous diversities, with 100,000 levels per environment [24]. Therefore, the generalization capability of agents needs to be measured by training on limited observations and performing gameplay in unfamiliar situations using ProcGen. To observe the effect of robustness contributed by ADAT, the performance trends of naive PPO and PPO supported by ADAT were compared during 40M timesteps. Bigfish and plunder Games, which possess a lot of visual information irrelevant to gameplay, and have few objects essential to control, were selected as the experimental environments. Both agents were trained at 200 levels and evaluated at 100,000 levels. Fig 5 can summarize contributions of the proposed auxiliary task in Bigfish and Plunder as:

- With the help of an action-driven auxiliary task, the performance of the PPO agent in the newly encountered environment has been dramatically improved. Furthermore, in Plunder Game, while vanilla PPO agent started to be saturated after 20M interactions, the agent with intrinsic representation progressively explored better policies.
- In the gameplay of Bigfish, a degradation of accomplishments in unseen levels game stood out clearly. It can be interpreted as the Bigfish Games demand hard generalization. Whereas the PPO agent underperformed apparently in few-shot levels, the agent aided by our novel auxiliary task coped well with unknown diversity inherent in the environment.

### Visual understanding with saliency map

In the above experiment, it was empirically shown that the agent gained sample-efficiency and generalization through ADAT. In this subsection, the saliency map experiment with a variety of detailed visual information was conducted to obtain insights into which pixels of image inputs the agent is focusing on. We measured the pixel-interest of the agent as to how much will the policy change if pixel-information is removed from the area around the location (*i*, *j*) [14].

$$\begin{array}{c} S_{V^{\pi }}\left(s,i,j\right)=\frac{1}{2}∥V^{\pi }\left(s\right)-V^{\pi }\left(\Phi \left(s,i,j\right)\right)∥{}^{2}. \end{array}$$ (5)

Saliency metric is the squared difference between the value estimate of the original sequence and the perturbed one. *Φ*(*s*, *i*, *j*) means the perturbation on image S at pixel coordinates (i,j). It removes pixel information by masking out a 5x5 size black patch around the (i,j) coordinate.

**Fig 6:**
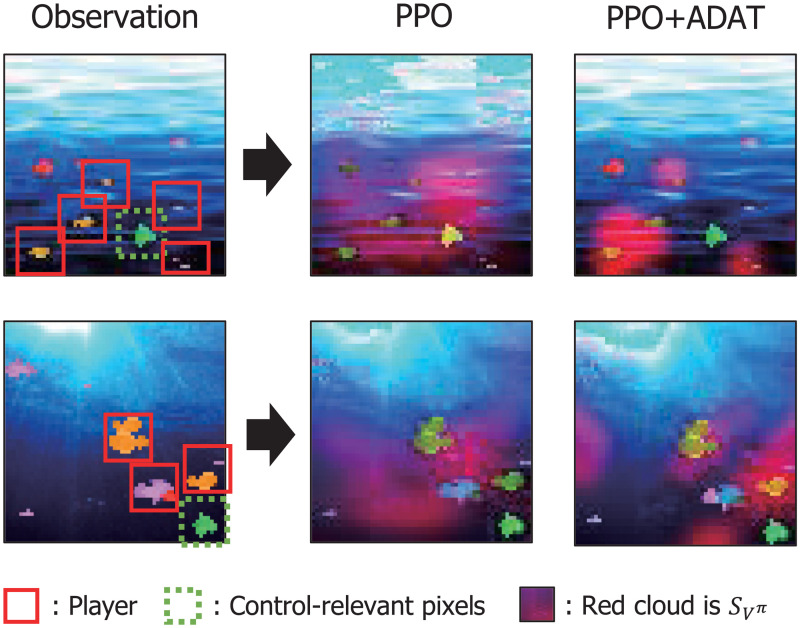
Saliency map of Bigfish and Plunder Games in OpenAI Progen. From left, original rendering, saliency map of the PPO, and the PPO with ADAT in order.

To demonstrate if the agent captures essential pixels for the control by performing our auxiliary task, we took a saliency map of PPO and ADAT PPO agents. Similar to the method in [14], pixels of the image were added to the R-value of RGB according to the normalized saliency score of the corresponding coordinates. As shown in Fig 6, the saliency map indicates an apparent difference between the PPO agent and the ADAT PPO agent. The Bigfish environment comprises a few crucial pixels for gameplay, so these pixels need to be focused. In the PPO agent map, the saliency score is evenly spread throughout on the whole input images. Whereas in ADAT, red cloud points are clustered around objects that the player fish should consider immediately for gameplay. These results visually validate that the pixels considered for inference were different between two agents through ADAT. Without the support of ADAT, the PPO agent became dependent to control-irrelevant details. This phenomenon made the agent vulnerable to the unnecessary characteristics of the environment. On the other hand, the ADAT PPO agent gained intrinsic representation capturing which pixels are crucial for addressing reinforcement learning problems and became independent to irrelevant details.

## Conclusion

In this work, we proposed ‘Action-Driven Auxiliary task,’ novel instance discrimination in a self-supervised manner, for representation to capture intrinsic features directly related to deciding actions and become insensitive to irrelevant details. Learning the shared structure between aggregated observations by contrastive representation, the agent distinguished control-irrelevant pixels and gained both sample-efficiency and generalization capabilities. These improvements are enhanced through proposed Unbiased Sampling. Our experiments on Atari and ProcGen demonstrated the efficacy of the ADAT and Unbiased Sampling module, visually confirming these intuitions. ADAT is a simple module attachable to various existing RL algorithms, both off-policy and on-policy. It is worthwhile investigating how to label continuous actions for pseudo-supervision as a future topic. In addition, in an environment where there are many tiny objects for control, such as Starpilot and Bossfight in OpenAI ProcGen, both existing and our representation methodologies have adversely affected the performance. Therefore, future work will be needed to make learning representation effective in these particular cases. d ac ipsum eget enim egestas ullamcorper nec euismod ligula. Curabitur fringilla pulvinar lectus consectetur pellentesque.
